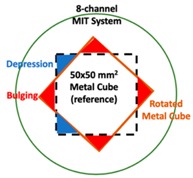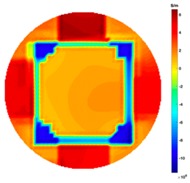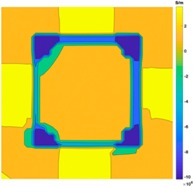# Monitoring Surface Defects Deformations and Displacements in Hot Steel Using Magnetic Induction Tomography †

**DOI:** 10.3390/s19133005

**Published:** 2019-07-08

**Authors:** Fang Li, Stefano Spagnul, Victor Odedo, Manuchehr Soleimani

**Affiliations:** 1 Engineering Tomography Lab (ETL), University of Bath, Bath, BA2 7AY, UK; 2 Product Division, Ergolines lab s.r.l., Area Science Park, Bldg. R3 Padriciano, Trieste 34149, Italy

**Keywords:** Magnetic Induction Tomography, imaging defects, imaging deformations, total variation algorithms, threshold-differencing algorithms, continuous casting

## Abstract

Magnetic Induction Tomography (MIT) is a non-invasive imaging technique that has been widely applied for imaging materials with high electrical conductivity contrasts. Steel production is among an increasing number of applications that require a contactless method for monitoring the casting process due to the high temperature of hot steel. In this paper, an MIT technique is proposed for detecting defects and deformations in the external surfaces of metal, which has the potential to be used to monitor the external surface of hot steel during the continuous casting process. The Total Variation (TV) reconstruction algorithm was developed to image the conductivity distributions. Nonetheless, the reconstructed image of the deformed square metal obtained using the TV algorithm directly does not yield resonable images of the surface deformation. However, differential images obtained by subtracting the image of a perfect square metal with no deformations from the image obtained for a deformed square metal does provide accurate and repeatable deformation information. It is possible to obtain a more precise image of surface deformation by thresholding the differential image. This TV-based threshold-differencing method has been analysed and verified from both simulation and experimental tests. The simulation results reported that 0.92% of the image region can be detected, and the experimental results indicated a 0.57% detectability. Use of the proposed method was demonstareted in a MIT device which was used in continuous casting set up. The paper shows results from computer simulation, lab based cold tests, and real life data from continoeus cating demonstating the effectiveness of the proposed method.

## 1. Introduction

Magnetic Induction Tomography (MIT) is a non-invasive and contactless imaging technique that is used to display the images describing passive electromagnetic properties (PEP), i.e., conductivity, permeability and permittivity [1,2]. The fundamental principle of MIT can be explained by the mutual inductance and eddy current theory: injecting an alternating current through excitation coils to generate a primary magnetic field, which interact with a conductive sample to induce a secondary magnetic field that can be detected by the sensor coil [3,4]. MIT has typically focused on imaging conductivity distributions of materials with high electrical conductivity by modelling the eddy currents in the forward model and then acquiring the conductivity distribution by solving the inverse problem [5]. The inverse problem in MIT is a major challenge, but this has been solved conventionally by The Tikhonov [3] and Total Variation (TV) [6] regularization algorithms. However, previous works have demonstrated that the Tikhonov regularization method may produce overly smoothed reconstructed images with blurred boundaries between different materials. The quality of the reconstructed images can be improved by an enhanced inverse solver, such as the Total Variation regularization technique [7,8].

In recent years, MIT has been widely proposed as an appropriate imaging technique in both industrial and medical applications, such as brain stroke detection [5], molten metal flow monitoring [9,10,11], pipelines inspection [12,13], multi-phase flow imaging [14] and non-destructive testing (NDT) for material characterization [15]. It’s well known that steel production has been attracting an increasing level of interest for potential use in various applications that require a contactless method for remotely monitoring the casting process. Many previous works [9,10,11] have sought to monitor the molten steel flow by reconstructing the conductivity distributions of the internal area of interest. However, MIT presents difficulties in detecting and imaging external surface defects and deformations due to its low spatial resolution. The inspection of the outer surface of pipelines using MIT was analyzed in [12] through a traditional MIT pixel-based reconstruction method (PBRM) and a narrowband pass filtering method (NPFM). Metal samples in a cylindrical shape with different types of damage at the outer surface have been investigated as defected pipelines using an 8-channel MIT system, which demonstrated that PBRM can retrieve information with a detectability of 10%, while NPFM can achieve a resolution of 2%.

This paper focuses on detecting the defects or deformations on the external surface of metallic targets using a Total variation algorithm-based MIT. This novel algorithm is described in [16]. Here, several simulation and experimental tests were carried out to investigate the ability of the novel algorithm to identify and image deformations and defects in metal. The proposed TV-based threshold-differencing algorithm was compared to the TV reconstruction method, and significant improvements were observed, which indicates that the novel algorithm for MIT may provide a suitable and valuable method for monitoring surface defects, deformations and displacements in hot steel continuous casting processes.

## 2. Methodology

The forward problem in MIT for surface defect application is based on the laws of induction and the eddy currents which are induced in the magnetic field with an alternating current [3]. The governing equation can be written as Equation (1):

(1) $$\nabla \times \frac{1}{\mu }\nabla \times A+j\omega \sigma A=J_{s}$$

where μ is the permeability, ω is the angular frequency, A is the total magnetic vector potential as a result of the current source $J_{s}$ and the effect of eddy current induced by the electrical conductivity σ. The current density can be determined by the magnetic vector potential according to the Biot-Savart Law. Equation (1) is solved by approximating the system as a combination of linear equations in small elements with appropriate boundary conditions using the Galerkin’s approximation [4,7]:

(2) $$\int_{\Omega_{all}}\left(\nabla \times N_{i}\cdot \frac{1}{\mu }\nabla \times A\right)dv+\int_{\Omega_{all}}(j\omega \sigma N_{i}\cdot A)dv=\int_{\Omega_{s}}\left(\nabla \times N_{i}\cdot T_{s}\right)dv$$

where $N_{i}$ is the linear combination of edge shape functions, $\Omega_{S}$ is the current source region (excitation coil), $\Omega_{all}$ is the entire region (current source region and eddy current region), $T_{s}$ is the electric vector potential, which is defined as:

(3) $$J_{s}=\nabla \times T_{s}$$

Then, after applying the volume integration equation, the induced voltage in the measuring coil can be calculated:

(4) $$V_{mn}=-j\omega \int_{\Omega_{s}}\left(A\cdot J_{0}\right)dv$$

where $J_{0}$ is the unit current density passing through the coil. 

The Jacobian matrix J can be expressed by the relationship between the induced voltage in the sensing coil and the conductivity:

(5) $$J=\frac{\partial V_{mn}}{\partial \sigma_{x}}=-\omega^{2}\frac{\int_{\Omega_{x}}A_{m}\cdot A_{n}dv}{I}$$

where $\sigma_{x}$ is the conductivity of pixel x, $\Omega_{x}$ is the volume of the perturbation, $A_{m}$ is the forward solver of excitation coil m excited by I and $A_{n}$ is the forward solver of sensor coil excited by unit current.

The inverse problem in MIT is defined as the retrieval of the unknown conductivity distributions σ of targets from the measured voltage $V_{measured}$, expressed by the linear equation:

(6) Δv=JΔσ

where $\Delta \sigma =\;\sigma -\sigma_{0}$, $\Delta v=V_{measured}-F\left(\sigma_{0}\right)$, *F* is the forward operator, $F\left(\sigma_{0}\right)$ means the initial estimate voltage obtained from forward problem, $\sigma_{0}$ is the initial estimate conductivity, J is the Jacobian matrix obtained from forward problem. The resolution of the reconstructed images can be improved by increasing the size of Jacobian matrix.

The inverse problem can then be represented by solving the least-square problem:

(7) $$\sigma_{\alpha }=argmin_{\Delta \sigma }({||J\Delta \sigma -\Delta v||}^{2})$$

The theory and application of both the Tikhinov and TV inverse solvers have been investigated and explained detailed in [7]. The total variation method can be defined by adding a penalty term to Equation (7):

(8) $$\sigma_{\alpha }=argmin_{\Delta \sigma }({||J\Delta \sigma -\Delta v||}^{2}+\alpha {||\nabla \;\Delta \sigma ||}_{1})$$

where α is the regularization parameter, ∇ is the gradient and ${||\cdot ||}_{1}$ is the $l_{1}-norm$. The anisotropic version of the discrete TV functional [6,8] is adopted in this paper:

(9) $$\alpha {||\nabla \;\Delta \sigma ||}_{1}=\alpha_{x}{||\nabla_{x}\;\Delta \sigma ||}_{1}+\alpha_{y}{||\nabla_{y}\;\Delta \sigma ||}_{1}+\alpha_{z}{||\nabla_{z}\;\Delta \sigma ||}_{1}$$

It should be mentioned that in this study, $\alpha_{x}=\alpha_{y}=\alpha_{z}=0.1$.

Then, the difficulty is to solve the constrained optimization problem:

(10) $$x_{\alpha }=argmin_{\Delta \sigma }\alpha {||\nabla \;\Delta \sigma ||}_{1}\;such\;that\;{||J\Delta \sigma -\Delta v||}^{2}<\rho$$

The above constrained optimization problem can be controlled by applying the Split Bregman iteration, which is an iterative method based on Bregman distance [8]. Then, the problem may be transferred to solve the Split Bregman equations [7]:

(11) $$\Delta \sigma^{k+1}=argmin_{\Delta \sigma }\frac{1}{2}{||J\Delta \sigma -\Delta v^{k}||}^{2}+\frac{\beta }{2}{||d^{k}-\nabla \Delta \sigma -b_{d}^{k}||}^{2}$$

(12) $$d^{k+1}=argmin_{d}\alpha {||d||}_{1}+\frac{\beta }{2}{||d-\nabla \Delta \sigma^{k+1}-b_{d}^{k}||}^{2}$$

(13) $$b_{d}^{k+1}=b_{d}^{k}+\nabla \Delta \sigma^{k+1}-d^{k+1}$$

where k is the $k^{th}$ iteration and d is an auxiliary variable.

Based on [16], the TV algorithm seeks to achieve the conductivity distribution of a perfect target $\Delta \sigma_{ref}$ which retains as reference data. Consequently, the conductivity distribution of the target contain a deformation, $\Delta \sigma_{def}$, which can be obtained using the TV method as well. Subtracting the reference conductivity distribution from the target conductivity distribution gives the deformed target conductivity as:

(14) $$\Delta \sigma_{diff}=\Delta \sigma_{def}-\Delta \sigma_{ref}$$

The image of the deformed target conductivity distribution was expected to indicate the location and the deformation information. And it’s obvious that the negative differential conductivity values represent the depression deformation part, and that the positive differential conductivity values represent the bulging. However, the reconstructed image of the deformed target is inadequate. For instance, the differential reconstructed image for a metal square with a depression deformation on one side may also show a bulging deformation on the opposite side due to the ill-posed nature of the inverse problem. This mirror effect can be tackled by comparing the absolute value of the maximum differential conductivity of deformed target |max{$\Delta \sigma_{def}$}| to the absolute value of the minimum differential conductivity |min{$\Delta \sigma_{def}$}|, and then eliminating the minimum to obtain the threshold-differential conductivity distribution, defined as [16]

(15) $$\Delta \sigma_{thres}=\begin{array}{ccc} \left\{\begin{array}{ccc} \Delta \sigma_{def} & \mathrm{when} & \Delta \sigma_{def}>0\\ 0 & \mathrm{when} & \Delta \sigma_{def}\leq 0 \end{array}\right\} & \mathrm{for} & \left|\max\left\{\Delta \sigma_{def}\right\}\right|>\left|\min\left\{\Delta \sigma_{def}\right\}\right|\\ \left\{\begin{array}{ccc} \Delta \sigma_{def} & \mathrm{when} & \Delta \sigma_{def}<0\\ 0 & \mathrm{when} & \Delta \sigma_{def}\geq 0 \end{array}\right\} & \mathrm{for} & \left|\max\left\{\Delta \sigma_{def}\right\}\right|<\left|\min\left\{\Delta \sigma_{def}\right\}\right| \end{array}$$

In fact, the quality and accuracy of deformed image can be further improved by forcing values below or above a certain threshold γ to zero, such that:

(16) $$\Delta \sigma_{thres}=\begin{array}{ccc} \left\{\begin{array}{ccc} \Delta \sigma_{def} & \mathrm{when} & \Delta \sigma_{def}>\gamma .\text{max}\left\{\Delta \sigma_{def}\right\}\\ 0 & \mathrm{when} & \Delta \sigma_{def}\leq \gamma .\text{max}\left\{\Delta \sigma_{def}\right\} \end{array}\right\} & \mathrm{for} & \text{abs}\left(\text{max}\left\{\Delta \sigma_{def}\right\}\right)>\text{abs}\left(\text{min}\left\{\Delta \sigma_{def}\right\}\right)\\ \left\{\begin{array}{ccc} \Delta \sigma_{def} & \mathrm{when} & \Delta \sigma_{def}<\gamma .\text{min}\left\{\Delta \sigma_{def}\right\}\\ 0 & \mathrm{when} & \Delta \sigma_{def}\geq \gamma .\text{min}\left\{\Delta \sigma_{def}\right\} \end{array}\right\} & \mathrm{for} & \text{abs}\left(\text{max}\left\{\Delta \sigma_{def}\right\}\right)<\text{abs}\left(\text{min}\left\{\Delta \sigma_{def}\right\}\right) \end{array}$$

where 0≤γ≤1. Introducing the γ will remove some amount of the noise data to make the results more stable and precise. After applying different values of γ during the research, we discovered that 0.3 ≤ *γ* ≤ 0.5 gave the optimal reconstructed images for the scenarios considered here. Appropriate values for γ give threshold-differential images that display the position of deformation individually. The complete images with deformation of the target can be obtained simply by adding the reference conductivity distribution to the threshold-differential conductivity distribution, such as:

(17) $$\Delta \sigma_{tot}=\Delta \sigma_{ref}+\Delta \sigma_{thres}$$

where $\Delta \sigma_{tot}$ is the total conductivity distribution. 

## 3. Simulation Results

**Figure 1:**
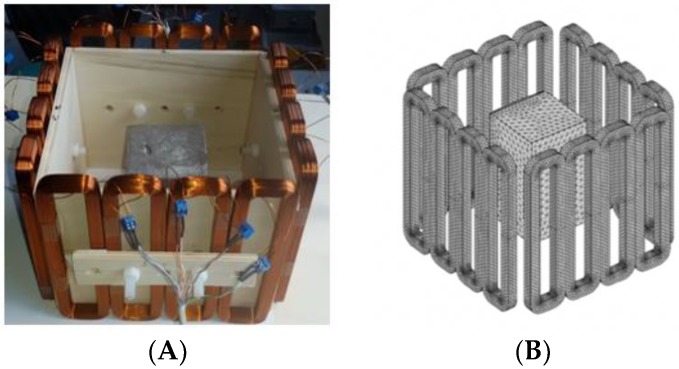
(**A**) An illustration of the laboratory prototype and (**B**) the simulation scenario.

In this section, we displayed the results in [16], which investigated the ability of the TV-based threshold-differential reconstruction method by reconstructing a 74 × 74 mm^2^ square sample with surface deformations using simulation data based on the laboratory prototype ( Figure 1A). The metal square sample was located at the center of a 16 coil array MIT system, as illustrated in Figure 1B, with an operational frequency of 130 Hz.

**Figure 2:**
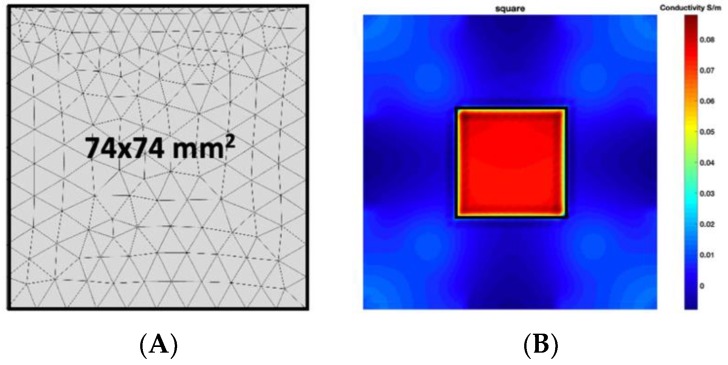
(**A**) Simulation setting of a perfect metal sample without any deformation and (**B**) Corresponding reconstructed image obtained by the simulation data.

Figure 2 shows the conductivity distribution obtained for a perfect square metal without any deformation using the TV reconstruction algorithm, which indicates that the algorithm can produce reconstructed images representing the square metal with reasonable accuracy. Then, bulging and depression deformation were considered.

**Figure 3:**
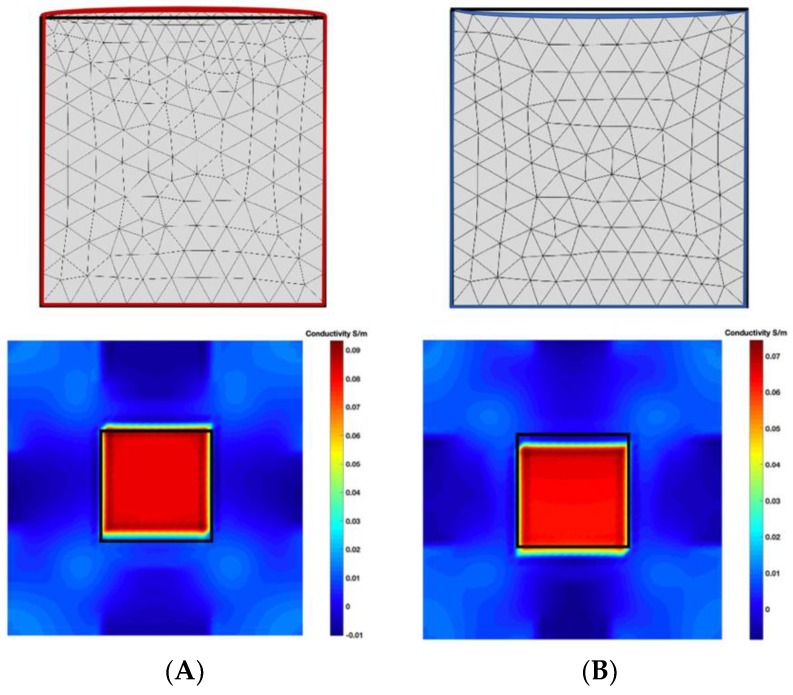
The simulation scenarios and the corresponding reconstructed images for (**A**) the metal with a surface bulge on top and (**B**) the metal with a surface depression on top, where the black line represents the actual location of a perfect square metal.

Figure 3 illustrates the simulation scenarios and the corrosponding reconstructed conductivity distribution images using TV algorithm for (A) a metal with a surface bulge on top, and (B) a metal with a surface depression on top, where the black lines represent the location of perfect square, the red line represents the boundaries of simulation setting of sample with bulging and the blue line represents the boundaries of sample with depression. The two scenarios were deformed by 9.25 mm at the midsection of one external surface. It’s obvious that although the bulge deformation in Figure 3A can be detected, the image may also be interpreted to include a depression at the bottom, as shown in Figure 3B.

**Figure 4:**
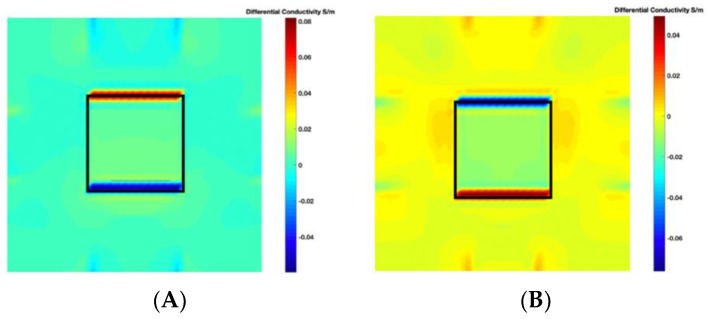
The differential conductivity distribution images obtained for a metal with (**A**) a bulge on top and (**B**) a depression on top.

Figure 4 illustrates the image obtained for the deformed target conductivity from applying Equation (14) for (A) the scenario with a bulge deformation on top, and (B) the scenario with a depression deformation on top, which therefore show only the effects of the deformation. It is obvious that a bulge (red) represented by positive numbers on the colorbar. A depression (blue) represented by negative numbers on the colorbar in both images can also be observed. However, the maximum and minimum values of $\Delta \sigma_{def}$ in the colorbar of the images shown in Figure 4 give a clue about the actual deformation in the considered scenario. In Figure 4A, the absolute value of the maximum deformed target conductivity, which represents a bulge, is greater than the absolute value of the minimum deformed target conductivity that represents a depression. Similarly, Figure 4B shows that the the absolute value of the minimum deformed target conductivity, which represents a depression, is greater than the absolute value of the maximum deformed target conductivity that represents a bulge.

**Figure 5:**
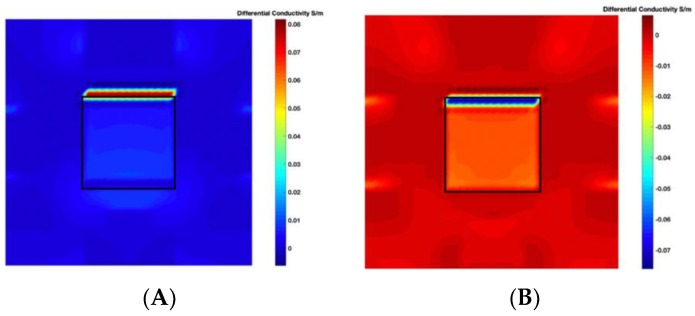
The threshold-differential conductivity distribution images obtained for (**A**) the metal with a bulge on top and (**B**) the metal with a depression on top.

The threshold-differential conductivity distribution can be plotted from Equation (15), obtained for the scenarios with deformation to get a more accurate representation of the particular deformation. Figure 5 shows the threshold-differential conductivity distribution obtained for (A) the metal with a bulge, and (B) the metal with a depression. Hence, a bulge represented by positive numbers on the colorbar in Figure 5A and a depression represented by negative numbers on the colorbar in Figure 5B can be observed, which indicates that we can obtain a better representation of the deformation in the scenario from $\Delta \sigma_{thres}$ than from $\Delta \sigma_{def}$.

**Figure 6:**
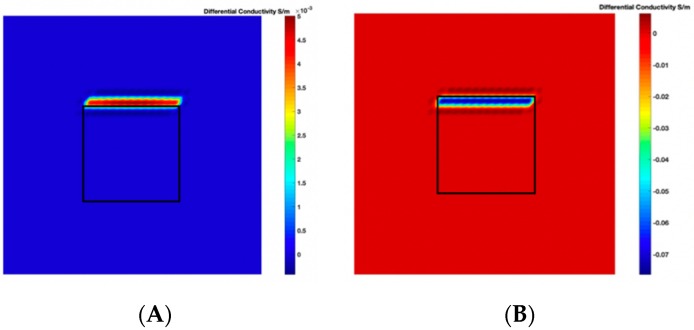
The threshold-differential conductivity distribution images obtained when γ=0.4 for (**A**) the metal with a bulge deformation on top and (**B**) the metal with a depression deformation on top.

By applying an appropriate value of γ to the Equation (16), the threshold-differential conductivity distribution images illustrate the location of the deformation alone. Figure 6 shows the threshold-differential conductivity distribution images obtained for (A) the metal with a bulge, and (B) the metal with a depression deformation when γ=0.4. The threshold-differential conductivity distribution images located the deformation more clearly by applying γ in Figure 6 than in Figure 5.

The TV-based threshold-differential image reconstruction algorithm with reference sample was analyzed for the outer shape reconstruction with surface deformations (bulging and surface depression defects), showing potential. Therefore, further research was conducted based on this method to analyze the image reconstruction of shapes with surface deformations. Supplementary simulation data were produced and analyzed: bulging or depression with different depth of the defect; bulging and depression at the same time on different surfaces and on the same surface.

**Figure 7:**
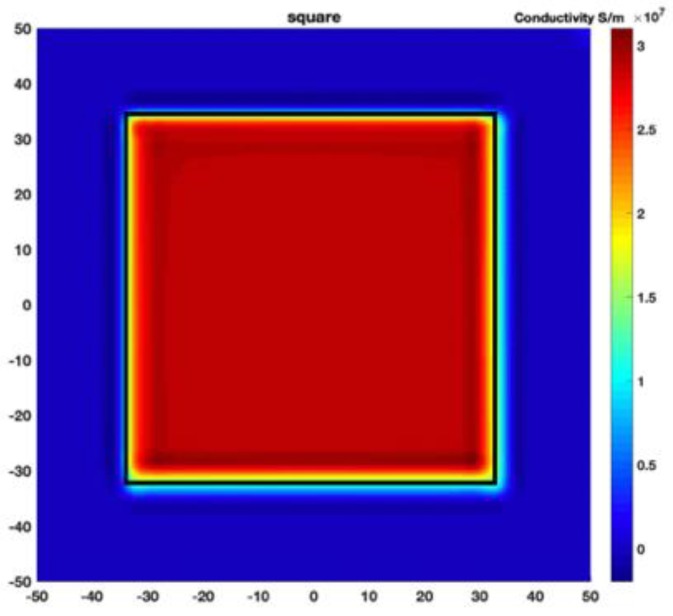
Reconstructed image for a square metal without any deformation.

Figure 7 shows the reconstructed image obtained for a square metal ($74\times 74\;{\mathrm{mm}}^{2})$ without any deformation using the TV reconstruction algorithm and larger Jacobian matrix (100×100). The resolution in this dissertation can be defined by a pixel resolution in pixel per mm. The size of the square in the reconstructed image is 64×64 pixels, which means that 1 pixel=1.156 mm; the resolution of this simulation scenario is 1/1.156=0.865 pixel/mm.

Samples with a bulging or depression defect on the top surface with a length of 74 mm (i.e., in the whole side) and with the 5 mm deformation depths were analyzed.

Table 1 indicates the threshold differential reconstructed images and their contour images obtained for the different forms of defects. The second column is the threshold differential image obtained for the sample with a bulging deformation on top with different defect depths. The fourth column is the threshold differential image obtained for the sample with depression deformation on top. The third and fifth columns are the corresponding contour images of reconstructed images. The blue line is the sample without any deformation, while the black lines are deformation parts. The threshold factor γ was set to 0.5. The iteration number used for solving TV function equals 500, in reference to Equations (7)–(9), which can provide reconstructed images with sharp boundaries.

The deformation detectability can be defined as:

(18) $$Deformation\;detectability=\frac{area\;of\;the\;detectable\;deformation}{area\;of\;the\;no\;deformed\;sample}$$

So, the deformation detectability of the proposed simulation system is $74\times 5/\left(200^{2}\right)=0.92\%$. In order to evaluate the accuracy of the proposed method applied to detect the surface defect, the normalized mean square error can be defined as [17]:

(19) $$err=\frac{{||\chi_{estimated}-\chi_{actual}||}^{2}}{{||\chi_{actual}||}^{2}}$$

where $\chi_{estimated}$ is the reconstructed defect depth value and $\chi_{actual}$ is the actual one. For the case in which bulging and depression deformations occur at the same time:

(20) $$err_{B+D}=\left(err_{Bulge}+err_{Depression}\right)/2$$

By referring to these quantitative metrics and the above results, the conclusion can be drawn that the value of the defect depth can be detected and acquired accurately with an error ($err_{Bulge\;deformation}=3.6\%$ and $err_{Depression\;deformation}=0.57\%$) through the proposed method. 

Table 2 illustrates the simulation scenario with two deformations at the same time (one bulging on the top surface and one depression at the bottom surface of the sample, both with a depth of 9.25 mm), as well as the differential reconstructed images. 

The results show that the TV-based differencing algorithm has the ability to detect two different types of deformation at the same time. By referring to Equations (19) and (20), the quantitative metrics err and the reconstructed defect depth can be acquired with an average error level of 1.45%.

Further simulation work was conducted on bulging and depressions at the same surface, which was achieved by transferring the square sample ($120\times 120\;{\mathrm{mm}}^{2}$) to a rhomboid one. The rhomboid samples can be defined by the parameter $R=\;\frac{Longest\;Diagonal\;length\;–\;Shortest\;Diagonal\;length}{Shortest\;Diagonal\;length}$. Table 3 illustrates the simulation scenario and the results of the rhomboid problem with R=0.5% and R=1.5%.

The positive values on the color bar represent bulging as well as the negative value mean depression. The conductivity values also give a clue of the amount of deformation. The results shows that the TV-based differencing algorithm has the ability to detect two deformations on the same surface.

## 4. Experimental Tests

**Figure 8:**
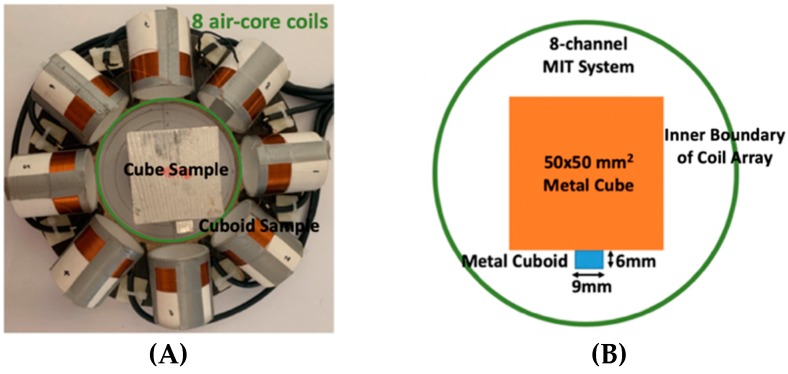
(**A**) Real experimental scenario of a metal cube and metal cuboid located at the center; (**B**) Illustration of the samples.

Regarding the inspection of pipelines, according to [12], simillar results can be obtained when testing the same damaged cylinder samples using the proposed algorithms. In this section, three sets of MIT machine experimental tests were conducted to investigate and display the ability of the threshold-differential algorithm to utilise the Total Variation (TV) reconstruction method to image a 50 × 50 mm^2^ metal cube with surface deformations located at the center of an 8-coil array, as illustrated in Figure 8. The MIT system in this research consists of (i) a host computer (ii) an National Instrument data acquisation system, and (iii) an equally-spaced eight x 50 turns coils with 40 mm diameter circular-array forming a 110 mm diameter imaging-region. The working frequency is 180 kHz. One metal cuboid represents the surface deformation. The demonstrated experiment system detectability is $6\times 9/\left(\pi \times 55^{2}\right)=0.57\%$. In this case:

(21) $$\Delta \sigma_{def}=\Delta \sigma_{cube+cuboid}$$

(22) $$\Delta \sigma_{ref}=\Delta \sigma_{cube}$$

Then, Equation (14) turns out to be:

(23) $$\Delta \sigma_{diff}=\Delta \sigma_{cube+cuboid}-\;\Delta \sigma_{cube}$$

Table 4 indicates the differential and threshold differential reconstructed images obtained for the sample with a bulging deformation at the center of different surfaces with different defect depths. The metal cuboid was placed at different positions which were close enough to the center of the metal cube surface. The two side lengths of the metal cuboid represent different defect depths (6 mm and 9 mm) according to different orientations. The threshold factor γ was set to 0.5. It can be determined that 1 pixel=2.08 mm, so the resolution of this experimental scenario is 1/2.08=0.48 pixel/mm.

The reconstructed images obtained by differential and threshold differential TV algorithms both precisely indicate the position of the defect. The threshold differential reconstructed images and the corresponding contour images shown in Column 3 and Column 4 demonstrate better image performance than the differential images displayed in Column 2. When compared to the results of Scenario 1 and 2, the threshold differential image shown in Row 2 has a longer reconstructed length of the bulging defect than that of the image shown in Row 1, which is consistent with the real experimental scenario. So, the results displayed above prove that the TV-based differencing algorithm has the ability to locate and reconstruct the bulging defect.

A second stage threshold can be adopted to refine the accuracy of position locating and depth detection by applying a larger number of γ to focus on the values around the maximum or minimum values and force the other values to zero. γ was set at 0.8 in this scenario. The metal cuboid sample was located at a different corner of the metal cube with a different orientation to achieve different positions and variable defect depths (6 mm and 9 mm). Table 5 displays the second stage threshold differential reconstructed images obtained for the sample with a bulging deformation in different corners with variable defect depths.

It’s obviously that the conductivity distribution images from the further threshold differential algorithm shown in Column 3 can locate and image the bulge more accurately than those obtained directly from the differential algorithm shown in Column 2. The defect depth can be acquired from the contour images displayed in Column 4 with an acceptable degree of error (1.7%, 0.08%, 0.9% and 1.5%).

Based on the simulation scenario with two deformation defects at the same time displayed in Table 2, the corresponding experimental tests were implemented by the displacement of the square sample. In this case, Equation (14) turns out to be:

(24) $$\Delta \sigma_{diff}=\Delta \sigma_{cube\;moved\;left\;or\;up}-\;\Delta \sigma_{cube}$$

Table 6 indicates the differential reconstructed images obtained for the sample with bulging and depression deformations at same time, which were achieved by moving the metal cube sample and then applying the abstraction. The red color in the differential reconstructed images, formed by positive numbers in the colorbar, and blue color parts, produced by negative numbers in the colorbar, represent the bulging and depression defect, respectively. 

The differential reconstructed images in Column 2 prove that the TV-based differencing algorithm can locate bulging and depression defects at the same time, as predicted from the simulation results shown in Table 2. It can be seen from the colorbar that the bulging amount is approximately the same as the depression amount, as it should be. Meanwhile, the reconstructed depth can be acquired by the corresponding contour images shown in Column 3, with a low level of error (5.1% and 11.1%). Moreover, the dispalcements of the sample can be detected by analyzing the differential resonstructed images.

Based on the simulation scenario of the rhomboid problem displayed in Table 3, the corresponding experimental tests were implemented by rotating the square sample with 45°. Table 7 indicates the differential reconstructed images obtained for the sample with bulging and depression deformations on the same surface, which were achieved by rotating the metal cube sample and then applying the subtraction. 

The red and blue color parts in differential reconstructed images represent the bulging and depression defects respectively. The results indicates that the TV-based differencing algorithm has the potential to reconstruct the two deformations at the same surface, but further research should be conducted to obtain more accurate results. 

## 5. Validation and Quantitative Evaluation of the Method 

The TV-based threshold-differential algorithm in MIT was adopted into real factory hot test data to track the movement of the strand during a continuous casting process. In this case:

(25) $$\Delta \sigma_{diff}=\Delta \sigma_{Time\_i}-\;\Delta \sigma_{referece}$$

where is $\Delta \sigma_{referece}$ is the conductivity distribution of the strand at one fixed time and $\Delta \sigma_{Time\_i}$ is any random time selected during the casting process.

**Figure 9:**
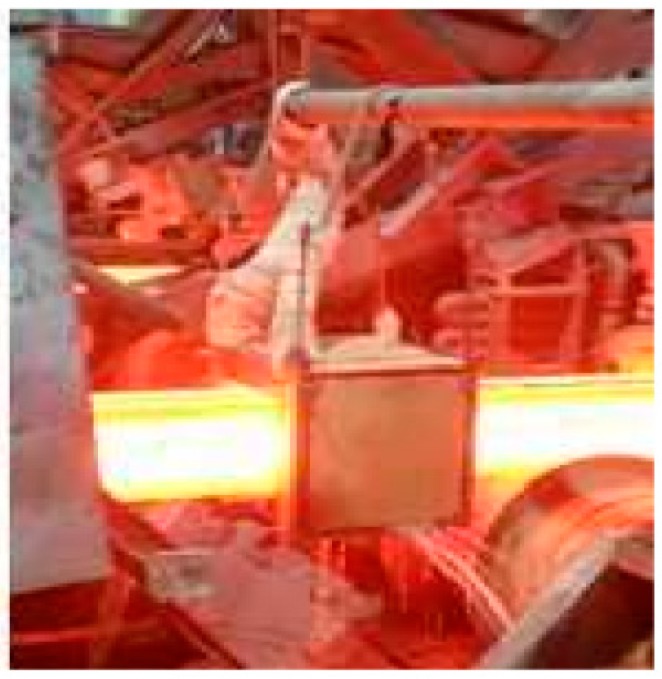
The MIT system in continuous casting set up.

Figure 9 shows the test set up for the 16 channel MTI system in the secondary stage of continuous casting. The system model is the same as coil geometry for Figure 1.

**Figure 10:**
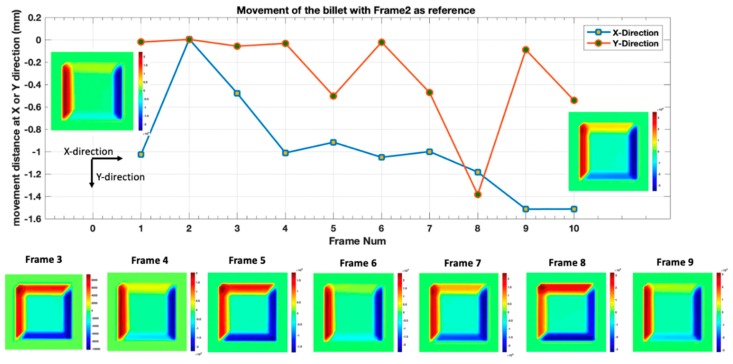
The movement direction and distance of the strand and its corresponding differential reconstructed images.

Figure 10 illustrates the movement distance of the strand in X- and Y-directions with its corrosponding differential reconstructed images. The positive number in the colorbar form the red colour part of the diffrential conductivity distribution, which indicated bulging and also the moving direction of the strand.

Moreover, two factors were defined to evaluate and validate the performance of the proposed method for monitoring the movement of the strand during a continuous casting process:

(26) $$pp=\frac{Sum\;of\;the\;differential\;conductivity\;distribution}{Sum\;of\;the\;conductivity\;distribution\;of\;reference\;Time}\times 100\;\;\;\left(\%\right)$$

(27) $$dis=\sqrt{{\left(Pos\_X\left(i\right)-Pos\_X_{reference}\right)}^{2}+{\left(Pos\_Y\left(i\right)-Pos\_Y_{reference}\right)}^{2}}\;\;\;\left(\mathrm{mm}\right)$$

**Figure 11:**
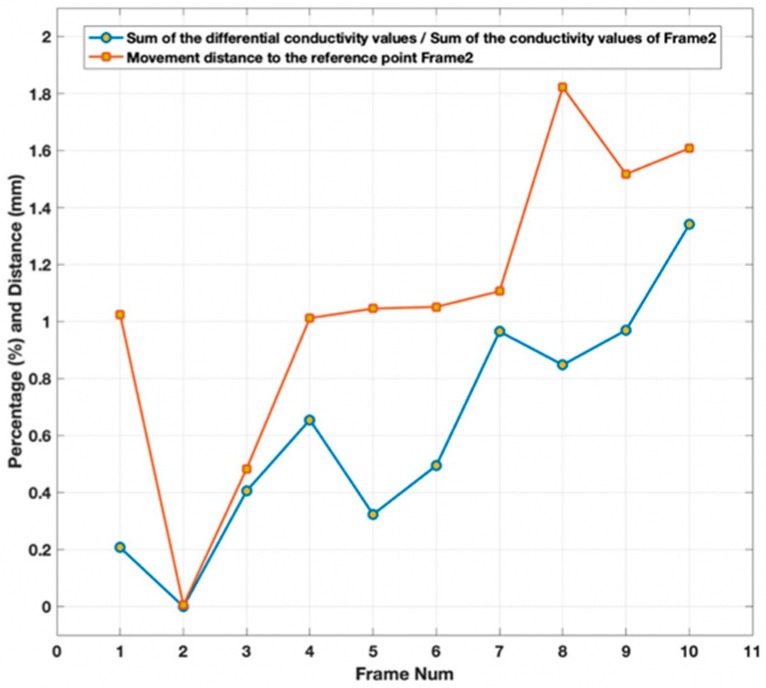
The movement distance of the strand to the reference point versus the sum values of bulging and depression with respect to the reference.

Figure 11 displays the relationship between the sum value of bulging and depression with respect to the reference, and the movement distance of the strand to the reference point. 

It is clear that the trend of these two plots is nearly the same, which indicates that the movement pattern of the strand is related to the sum conductivity distribution values of the differential reconstructed images. Therefore, the TV-based threshold-differential algorithm can be treated as a suitable method for monitoring surface defects, deformations and displacements.

## 6. Conclusions

A TV-based threshold-differential algorithm in MIT for surface defects and deformations is presented and validated using simulation and lab experimental tests as well as hot test in real continuous casting. The total conductivity distribution image obtained by simulations and experiments shows that the threshold-differential algorithm gives much better representation of bulge or depression deformation than that obtained from the TV method directly. The proposed simulation scenario with a resolution of 0.865 pixel/mm can achieve a deformation detectability of 0.92%. The proposed MIT experimental system can achieve a detectability of 0.57% with a resolution of 0.48 pixel/mm. The displacements of the sample can also be detected and located using the proposed method. The results presented in this paper confirm and prove the TV-based threshold-differencing method applied in MIT as a viable tool for monitoring and detecting surface defects, deformation and displacements in hot steel tests. The lab sample shown for the laboratory-based experiments conducted in this paper are the combination of two samples several more lab based experiments are conducted with success not included in here. The real process based data and results shows demonstartion of this new imaging method and system for contneous casting application. 

## Figures and Tables

**Table 1:** Threshold Differential reconstructed images for bulging and depression defects.

Defect Depth (Simulated)	Threshold Differential Image Obtained for the Square with a Bulge Deformation on Top	Defect Depth (Reconstructed)	Threshold Differential Image Obtained for the Square with a Depression Deformation on Top	Defect Depth (Reconstructed)
5 mm	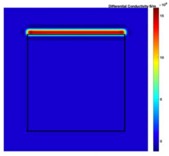	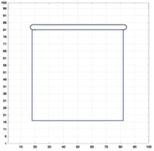 3.5 pixels = 4.046 mm	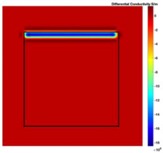	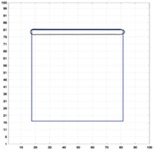 4 pixels = 4.624 mm

**Table 2:** Differential reconstructed images of two deformations at same time (Pixel = 100).

Scenarios	Differential Reconstructed Images	Contour Images	Defect Depth(Reconstructed)
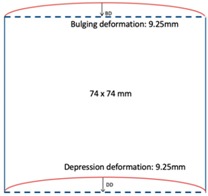	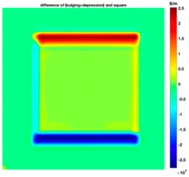	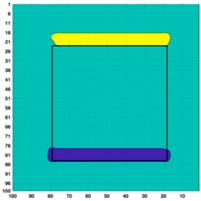	Bulging (top): 7 pixels = 8.092 mm $err_{Bulge\;}=1.6\%$ Depression (bottom): 7.1 pixels = 8.208 mm $err_{Depression\;}=1.3\%$

**Table 3:** Differential reconstructed images of two deformations at same surface (Pixel = 50).

Scenarios	Differential Reconstructed Images	Contour Images
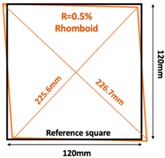	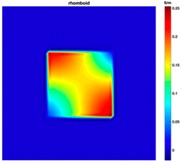	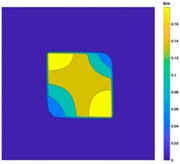
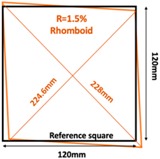	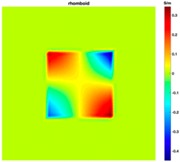	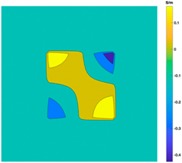

**Table 4:** Differential and Threshold Differential reconstructed images for a defect at the center of different surfaces with different depths (Pixel = 50).

Scenarios	Differential Reconstructed Images	Threshold Differential Reconstructed Images	Contour Images
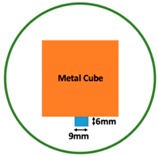	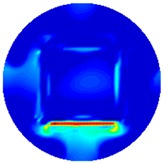	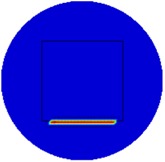	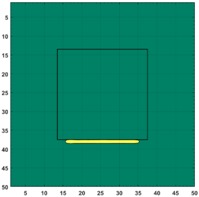
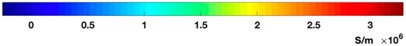			
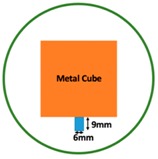	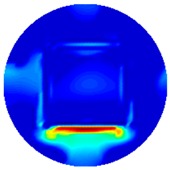	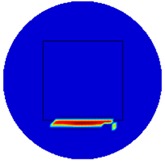	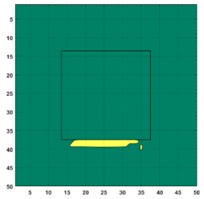
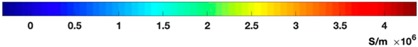			
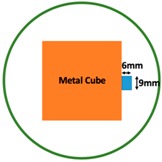	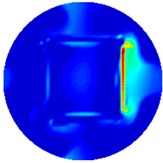	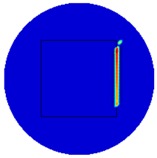	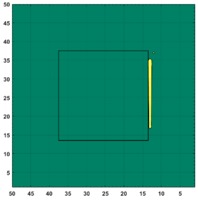
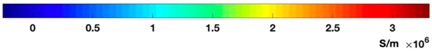			
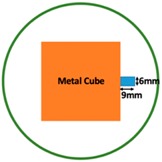	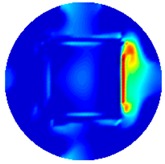	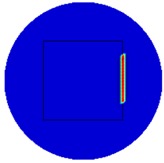	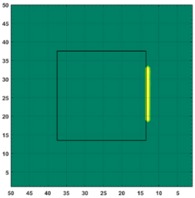
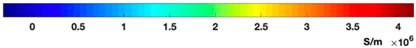			

**Table 5:** Differential reconstructed images refined by A second stage threshold for defects at different positions with different depths (Pixel = 50).

Scenarios(Actual Depth)	Differential Reconstructed Images	Reconstructed Images Refined by A second Stage Threshold	Contour Images(Reconstructed Depth)
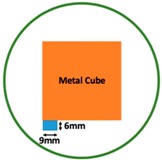	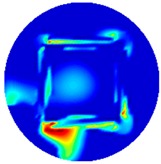	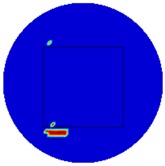	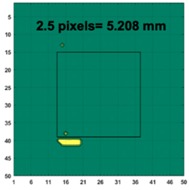 err=1.7%
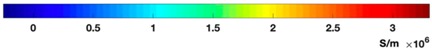			
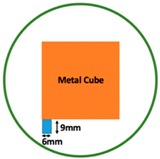	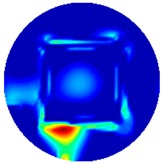	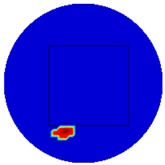	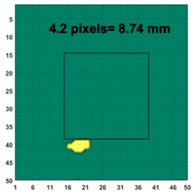 err=0.08%
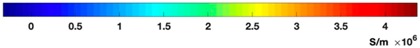			
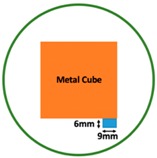	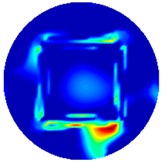	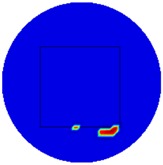	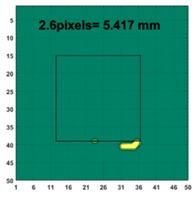 err=0.9%
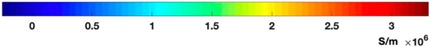			
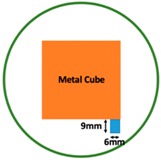	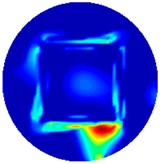	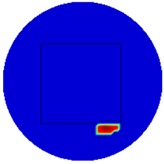	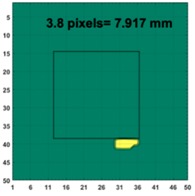 err=1.5%
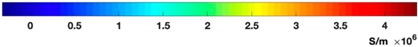			

**Table 6:** Differential reconstructed images for bulging and depression defects at same time (Pixel = 50).

Scenarios	Differential Reconstructed Images	Reconstructed Defect Depth	Contour Images
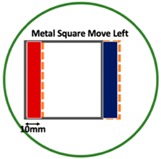	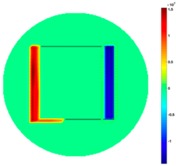	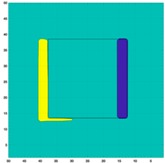	**Bulging (left)** **3.5 pixels = 7.292 mm** **Depression (right)** **4 pixels = 8.333 mm** $err_{B+D\;}=5.1\%\;$
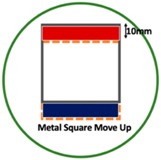	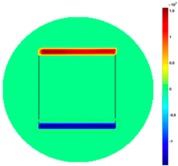	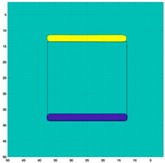	**Bulging (top)** **3.2 pixels = 6.667 mm** **Depression (bottom)** **3.2 pixels = 6.667 mm** $err_{B+D\;}=11.1\%$

**Table 7:** Differential reconstructed images for bulging and depression defects on the same surface (Pixel = 50).

Scenarios	Differential Reconstructed Images	Contour Images